# Peptidomic characterization of peptide processing in the hippocampus of Wfs1 knockout mice

**DOI:** 10.1186/2193-1801-4-S1-P48

**Published:** 2015-06-12

**Authors:** Karin Tein, Sergo Kasvandik, Eero Vasar, Anton Terasmaa

**Affiliations:** Institute of Biomedicine and Translational Medicine, University of Tartu, Estonia

**Keywords:** Peptide processing, Wfs1, pro-SAAS

Mutations in WFS1 gene cause Wolfram syndrome, which is a rare autosomal recessive disorder, characterized by diabetes insipidus, diabetes mellitus, optic nerve atrophy and deafness (DIDMOAD). WFS1 gene product wolframin is located in the endoplasmic reticulum. Mice lacking this gene have disturbances in processing and secretion of peptides, such as vasopressin and insulin. In the brain, high levels of wolframin protein are observed in the hippocampus, amygdala and limbic structures. The aim of this study was to investigate the effect of Wfs1 invalidation on the peptide processing in hippocampus of mice. Peptidomic approach was used to characterize individual peptides in the hippocampus of wild type and Wfs1 knock-out mice. We identified 126 peptides in the hippocampal extracts and levels of 10 peptides were different in Wfs1 and wild type mice at (P<0.05). Largest alteration was found in the level of peptide little-LEN, which is processed (cleaved) from pro-SAAS (Pcsk1n) in prohormone convertase 2 (PC2) dependent ways. Results of this study reveal alterations of peptide processing in the hippocampus of Wfs1 deficient mice.

